# Integrative Analysis of Widely Targeted Metabolomics and Flavoromics Reveals Dynamic Changes in Characteristic Metabolites at Different Processing Stages of Black Highland Barley Beer

**DOI:** 10.3390/molecules31173062

**Published:** 2026-08-31

**Authors:** Hong Wang, Wengang Zhang, Wancai Zheng, Bin Dang, Xijuan Yang

**Affiliations:** 1 Academy of Agriculture and Forestry Sciences, Qinghai University, Xining 810016, China; yb240901010040@qhu.edu.cn (H.W.); 2017990098@qhu.edu.cn (W.Z.); 2021990018@qhu.edu.cn (W.Z.); 2008990019@qhu.edu.cn (B.D.); 2 Key Laboratory of Qinghai Province Tibetan Plateau Agric-Product Processing, Qinghai University, Xining 810016, China; 3 Laboratory for Research and Utilization of Qinghai Tibet Plateau Germplasm Resources, Qinghai University, Xining 810016, China

**Keywords:** black highland barley, beer, processing stages, flavoromics, widely targeted metabolomics

## Abstract

Black highland barley, rich in phenolic compounds and β-glucans, is increasingly used in craft beer brewing, but the dynamic metabolic changes across its processing chain remain unclear. This study integrated GC×GC-TOF MS based flavoromics with UHPLC-QTRAP-MS based widely targeted metabolomics to track volatile and non-volatile metabolites from raw grain (K1) through germinated malt (K2) and roasted malt (K3) to finished beer (K4). A total of 256 volatile compounds and 615 non-volatile metabolites were identified. Alcohols and hydrocarbons dominated K1. Germination (K2) enriched aldehydes and reduced esters by 66%. Roasting (K3) generated Maillard reaction products, including 2,3,5-trimethylpyrazine (198.4-fold increase) and furfural (20.2-fold increase). Fermentation (K4) shifted the profile to ester dominance (40.95%), with isoamyl alcohol (ROAV = 71.59) and 2-undecanone (ROAV = 65.37) as the principal aroma contributors. Notably, 94.1% of phenolic and flavonoid compounds (168 of 184) exhibited higher abundance in finished beer than in raw grain. Their levels declined after roasting and then accumulated during fermentation. Cross-omics correlation indicated a strong association between furfural and its pentose phosphate precursor (ρ = 1.00, *p* < 0.001). These findings demonstrate that fermentation acts as the decisive metabolic turning point that simultaneously shapes the flavor profile and nutritional quality of black highland barley beer. The finished beer contained 250.45 mg GAE/L total phenolics and 97.91 mg RE/L total flavonoids, with ABTS and DPPH radical scavenging rates of 87.41% and 92.68%, respectively.

## 1. Introduction

Highland barley (*Hordeum vulgare* L. var. nudum), also known as naked barley or “Qingke” in Chinese, is a hull-less cereal adapted to the Qinghai–Tibet Plateau. Among its color variants, black highland barley, characterized by a black-purple pericarp, has been cultivated in this region for millennia and is well adapted to harsh high-altitude environments [1]. This black-grained variety is particularly rich in bioactive constituents, including β-glucans, phenolic compounds, dietary fibers, and amino acids [2]. A comparative analysis revealed that the black-grained barley had a total phenolic content of 512.38 mg/100 g DW, higher than the 457.02 mg/100 g DW found in yellow-grained barley. Its bound phenolic content was 241.62 mg/100 g DW, whereas that of the yellow variety was 191.40 mg/100 g DW, with the differences being statistically significant [3]. Additionally, black highland barley contains 18 amino acids, including all eight essential ones, with glutamate being the most abundant at 2.67% [4]. These bioactive compounds have been associated with antioxidant, anti-inflammatory, and hypoglycemic effects. Such compositional features align with modern consumer demand for healthy foods and underscore its promising potential for functional food development [5,6]. The industrialization of black highland barley has accelerated significantly in recent years. In Longzi County, Tibet, certified as the “world’s largest black highland barley planting base” in 2023, the cultivation area has expanded from 867 ha in 2014 to 2033 ha, with major processing enterprises achieving an output value of CNY 29.3 million in 2024; black highland barley beer alone generated over CNY 10 million in annual sales. In Garzê Prefecture, Sichuan Province, the planting area reached 9333 ha in 2024, while in Nangqên County, Qinghai Province, 1866 ha are under cultivation, with products securing CNY 20 million in intent purchase orders at the 2023 China Food and Drinks Fair [7,8,9,10]. These data demonstrate that black highland barley has evolved into a high-value specialty agricultural product with broad application prospects in functional foods and craft beers.

Beer quality is shaped by multiple factors, including raw material composition, enzymatic transformations during malting, thermal reactions during roasting, and microbial metabolism during fermentation [11]. Among these, barley malt serves as the primary source of starch-derived fermentable sugars, nitrogenous nutrients, and flavor precursors [12]. In recent years, with the rapid growth of the craft beer market, there has been increasing interest in utilizing locally adapted cereal varieties—such as white sorghum, oats, buckwheat, and highland barley—to develop beers with distinctive flavor profiles and functional properties [13,14,15,16,17].

The core processes of beer production—germination, roasting, and fermentation—fundamentally shape malt quality and beer flavor [18]. Germination activates endogenous enzymes (amylases, β-glucanases, lipases, and proteases) that degrade starch, β-glucans, lipids, and proteins into low-molecular-weight compounds [19], while also elevating bioactive components such as phenolic acids and flavonoids [20,21,22]. Roasting is critical for malt aroma development. During roasting, the main precursor groups are reducing sugars (e.g., ribose, fructose, and glucose), free amino acids (e.g., glycine, cysteine, and phenylalanine), and lipids. These compounds undergo Maillard reactions to form pyrazines, Strecker degradation to generate aldehydes, and lipid peroxidation to produce alcohols and ketones, collectively generating characteristic heterocyclic aromas and releasing bound phenolics [23,24,25]. Yeast fermentation subsequently converts sugars and amino acids into higher alcohols (e.g., isoamyl alcohol via the Ehrlich pathway) and branched-chain fatty acids, which are subsequently esterified with acyl-CoA to produce fruity and floral ester compounds such as isoamyl acetate and ethyl hexanoate, imparting fruity and malty notes to the final product [26].

Although black highland barley—rich in nutritional and functional components—is increasingly applied in beer brewing, existing studies have focused primarily on malt addition ratios or single processing stages, lacking a comprehensive dynamic analysis of metabolomics and flavoromics across the entire production chain. Black highland barley is particularly relevant to beer production due to its high content of bioactive phenolics and β-glucans, which can enhance the antioxidant capacity and mouthfeel characteristics of the finished beer. However, compared with conventional malting barley, black highland barley (a hull-less variety) exhibits distinct germination behaviors, enzymatic activities, and roasting responses, which may affect lautering efficiency and final flavor complexity [11,27]. To bridge the gap between raw material characteristics and final product quality, this study tracked metabolic changes across four representative stages of black highland barley beer production: raw grain (K1), germinated malt (K2), roasted malt (K3), and finished beer (K4).

To date, most studies on barley, malt, and beer have employed either a single analytical platform or focused on isolated production stages, limiting coverage of both volatile and non-volatile metabolites across the full production chain. In contrast, this study integrates flavoromics (GC×GC-TOF MS) with widely targeted metabolomics (UHPLC-QTRAP MS) to simultaneously characterize metabolites across K1–K4. This is further supplemented by ROAV-based aroma prioritization, cross-omics precursor correlation analysis, and phenolic trajectory tracking. To the best of our knowledge, this multi-dimensional workflow is the first to systematically map the aroma–metabolite relationship across the complete production chain of black highland barley beer from raw grain to finished product.

## 2. Results

### 2.1. Raw Material Characteristics of Black Highland Barley and Their Influence on Finished Beer Quality

Malt amino acid profile (mg·g^−1^): Asp 0.875, Thr 0.170, Ser 0.230, Glu 2.095, Gly 0.445, Ala 0.445, Cys 0.270, Val 0.325, Met 0.135, Ile 0.305, Leu 0.690, Tyr 0.155, Phe 0.265, His 0.415, Lys 0.455, Arg 0.265, Pro 0.565.

The raw black highland barley grain contained 9.22% protein, 58.76% total starch, and 3.66% β-glucan (Table 1). After malting, the protein content increased by 34.8% (to 12.43%), soluble dietary fiber increased by 224.5% (from 2.69% to 8.73%), and β-glucan decreased by 30.1% (to 2.56%)—changes favorable for lautering and beer clarity. The apparent increase in protein and ash content (from 1.56% to 2.10%) is mainly attributable to the concentration effect caused by dry matter loss during germination, as carbohydrates are consumed as respiratory substrates. The total amino acid content in the malt was 8.105 mg·g^−1^, with essential amino acids accounting for 34.0%. The Kolbach index of the malt reached 53.59%, indicating a high degree of protein modification, which may be suitable for certain specialty beer styles. These characteristics underpin the robust enzymatic activation and metabolic reprogramming observed during the germination stage.

After roasting, the roasted black highland barley malt exhibited an extract content of 72.87%, α-amino nitrogen of 144.40 mg/100 g, and saccharification power of 108.40 WK (Table 2). The saccharification power of 108.40 WK remained high, supporting efficient starch-to-sugar conversion in subsequent fermentation despite partial enzyme inactivation during roasting.

The finished black highland barley beer showed an alcohol content of 3.28%vol with a fermentation degree of 76.94%, reflecting efficient sugar-to-ethanol conversion by yeast. The original extract was 8.80 °P, consistent with the moderate gravity brewing of black barley beer, and the beer color reached 16.24 EBC, reflecting the characteristic dark appearance imparted by Maillard-derived melanoidins from roasting. Total acidity was 1.97 mL·100 mL^−1^ and pH was 4.30, consistent with the typical acidity profile of quality beer. Total phenolics were 250.45 mg GAE/L and total flavonoids were 97.91 mg RE/L. The ABTS radical scavenging rate was 87.41% and the DPPH radical scavenging rate was 92.68%, demonstrating strong in vitro antioxidant capacity of the finished beer.

### 2.2. Changes in Volatile Flavor Compounds of Black Highland Barley Malt with Processing Stage

#### 2.2.1. Comparison of Volatile Flavor Compound Composition Among Different Samples

The volatile components of black highland barley raw material (K1), germinated malt (K2), roasted malt (K3), and beer (K4) were obtained by GC×GC-TOF MS. As shown in Figure S1, the one- and two-dimensional ion chromatograms exhibited stable baselines, well-resolved peaks, and intact peak shapes, confirming the reliability of the analytical method.

**Figure 1:**
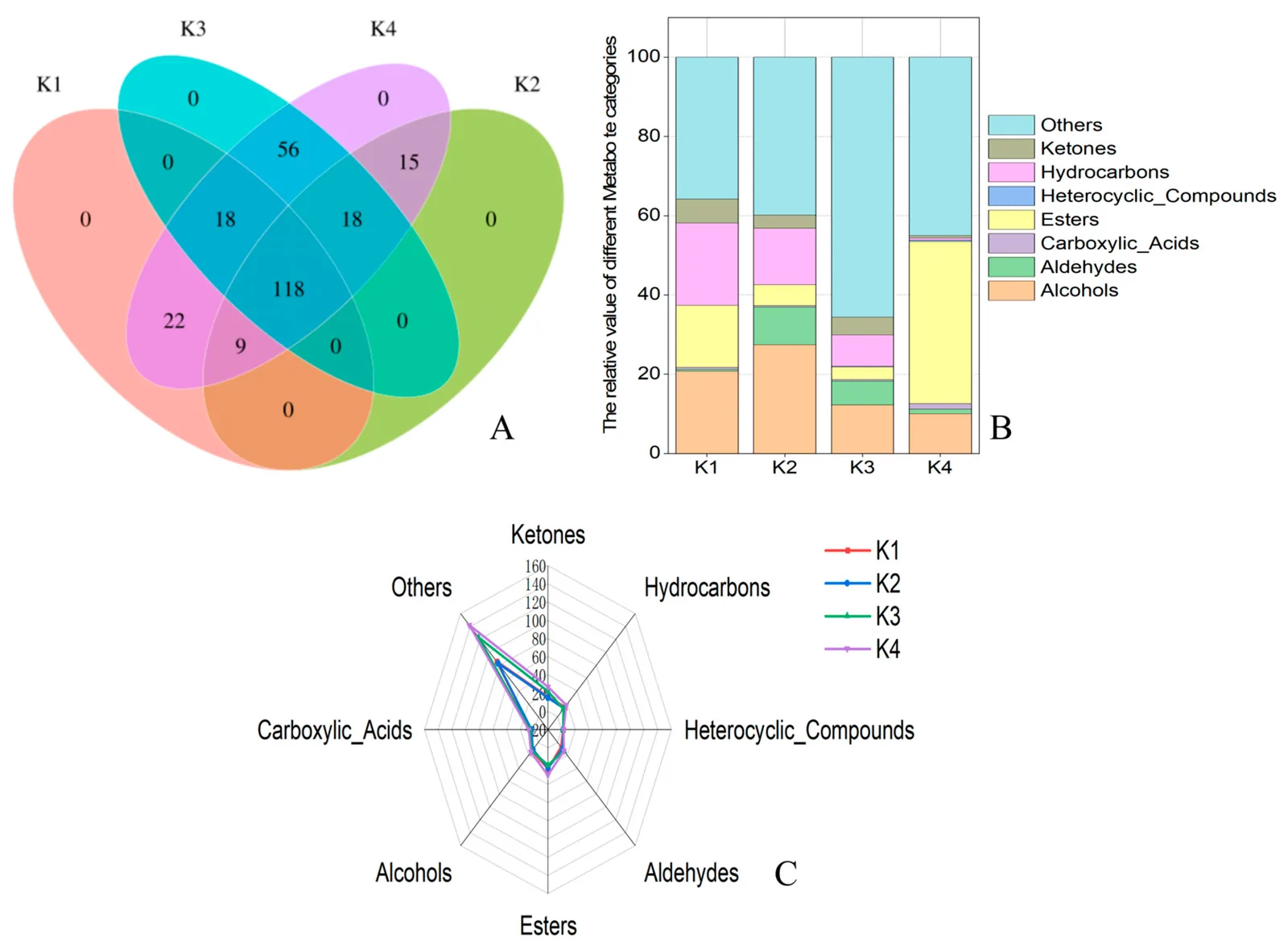
Composition of volatile flavor compounds in black highland barley malt at different processing stages. (**A**) Venn diagram; (**B**) content stacked chart; (**C**) classification radar chart. K1, K2, K3, and K4 represent black highland barley raw material, germinated malt, roasted malt, and beer, respectively. Data are expressed as mean ± SD (n = 3).

In total, 256 volatile compounds were annotated across the four stages, with 167, 160, 210, and 256 detected in K1, K2, K3, and K4, respectively (Figure 1A–C). As shown in Figure 1B, alcohols and hydrocarbons were the predominant classes in K1. In K2, the relative content of alcohols and aldehydes increased, while esters decreased by 66%. In K3, hydrocarbons and alcohols decreased, while heterocyclic compounds (e.g., pyrazines) increased. In K4, esters became the predominant class, accounting for 40.95% of total volatile compounds. Vanillin and pyrazines were detected across all four stages.

#### 2.2.2. Orthogonal Partial Least Squares-Discriminant Analysis (OPLS-DA)

**Figure 2:**
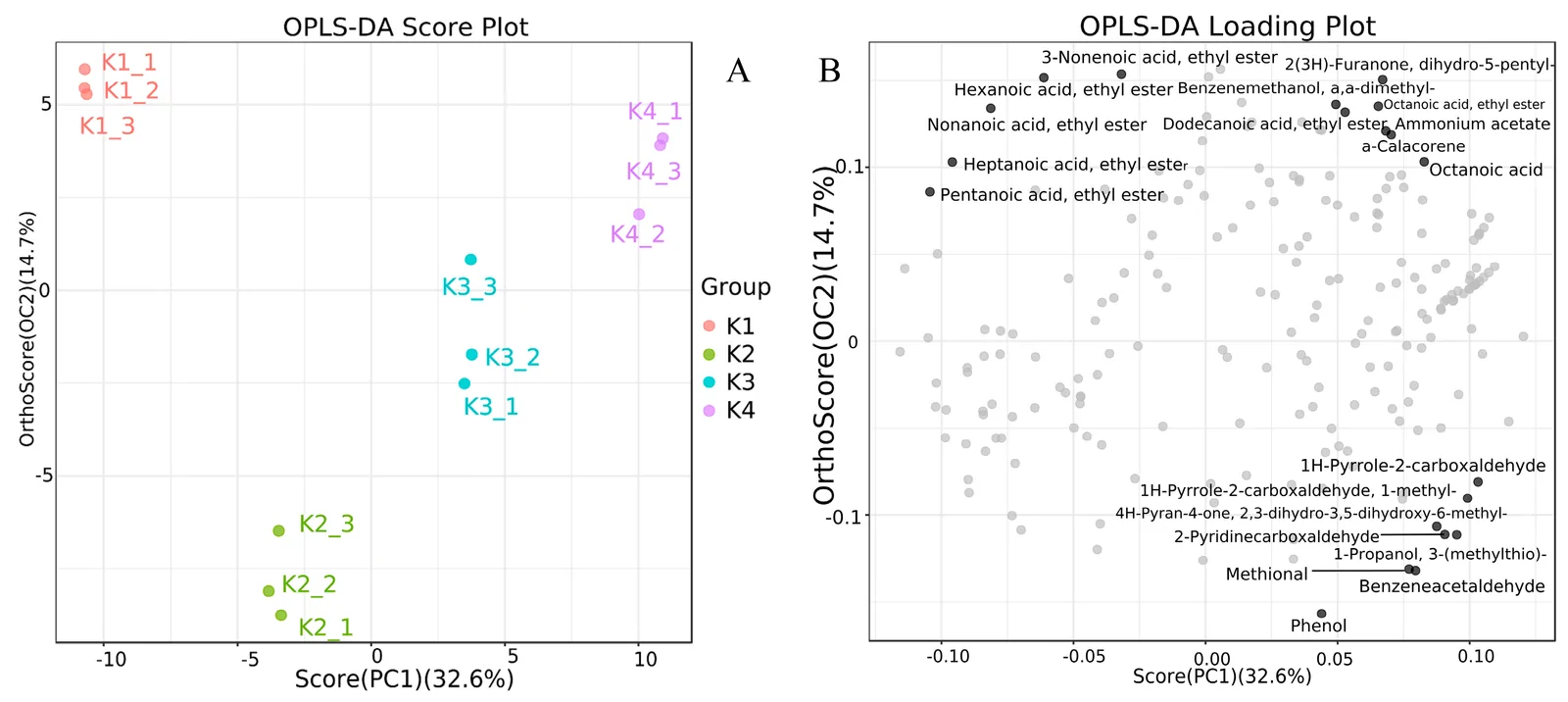
OPLS-DA of volatile flavor compounds across different processing stages. (**A**) OPLS-DA score plot; (**B**) OPLS-DA loading plot. K1, K2, K3, and K4 represent black highland barley raw material, germinated malt, roasted malt, and beer, respectively. Data are expressed as mean ± SD (n = 3).

The OPLS-DA score plot (Figure 2A) demonstrated that the first two principal components explained 47.3% of the cumulative variance (PC1: 32.6%, PC2: 14.7%). K1 and K2 formed a cluster on the negative PC1 semi-axis, whereas K3 and K4 were located on the positive PC1 semi-axis. The loading plot (Figure 2B) displayed the 20 volatile compounds contributing most to intergroup differentiation, with 5 volatile compounds located on the negative half-axis of PC1 and 15 on the positive half-axis.

#### 2.2.3. Analysis of Differential Volatile Flavor Compounds Among Different Samples

**Figure 3:**
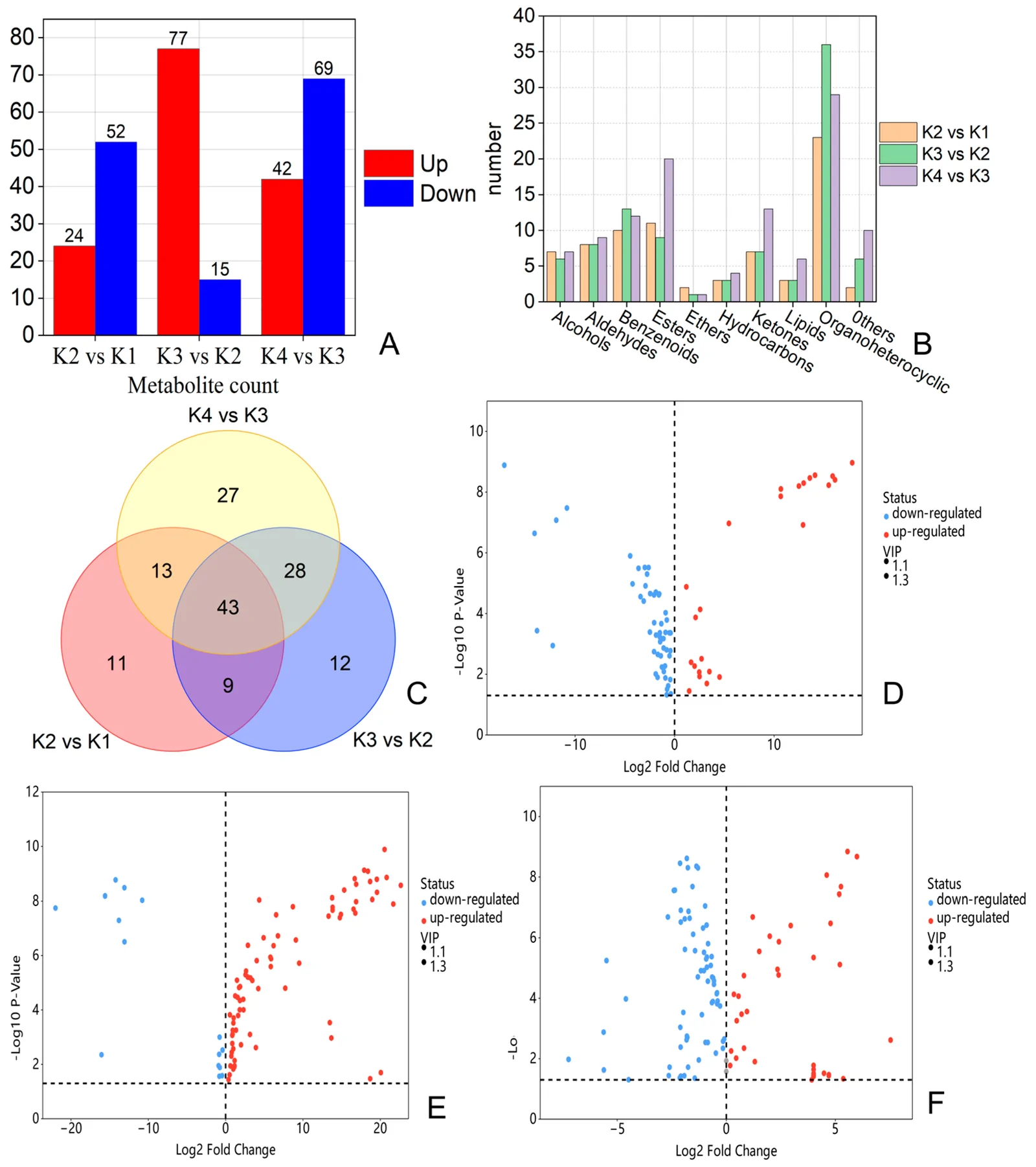
Analysis of differential volatile flavor compounds in black highland barley malt at different processing stages. (**A**) Quantity statistics; (**B**) category statistics; (**C**) overlapping Venn diagram; (**D**) K2 vs. K1, (**E**) K3 vs. K2, (**F**) K4 vs. K3 differential metabolite volcano plots. K1–K4 as defined in Figure 1. Data are expressed as mean ± SD (n = 3).

Under VIP > 1 and *p* < 0.05, 76 (K2 vs. K1), 92 (K3 vs. K2), and 111 (K4 vs. K3) differential volatile compounds were identified, with organic heterocyclics, aldehydes, and esters as the predominant classes (Figure 3A,B). Among these, 11, 12, and 27 unique differential compounds were observed in the three comparison groups, respectively, representing stage-specific aroma markers (Figure 3C).

As shown in Figure 3D, germination (K2 vs. K1) resulted in the downregulation of 52 volatile components, mainly alcohols, esters, and ketones, and the upregulation of 24 compounds, predominantly aldehydes such as nonanal (from 0.66% to 3.78%) and benzeneacetaldehyde (from 0.30% to 13.46%). The relative content of 1-octen-3-ol increased significantly from 8.02% to 18.16%.

Roasting (K3 vs. K2) significantly upregulated 77 volatile components, primarily Maillard reaction products (Figure 3E). Compared with K2, the relative contents of 2,3,5-trimethylpyrazine and furfural increased by 198.4- and 20.2-fold, respectively. Meanwhile, 1-octen-3-ol decreased by 44.14% (*p* < 0.01), and limonene was completely depleted.

During fermentation (K4 vs. K3), volatile components underwent further substantial transformation (Figure 3F). Forty-two compounds were upregulated, mainly esters (e.g., isoamyl acetate, ethyl butyrate) and terpenes (e.g., limonene). In contrast, 69 compounds were downregulated, including alcohols (e.g., 1-pentanol, 1-octen-3-ol), aldehydes (e.g., decanal, nonanal), and heterocyclics. Notably, the relative content of 1-octen-3-ol decreased substantially from 10.14% to 0.04%, and several harsh aldehydes (e.g., decanal, nonanal) also declined substantially.

#### 2.2.4. ROAV-Based Screening of Aroma-Active Compounds

To identify the key aroma contributors across the processing chain of black highland barley beer, the relative odor activity value (ROAV) method was employed, with vanillin as the reference standard. ROAVs were calculated for 77 volatile compounds (Table S1). Using ROAV ≥ 1 as the aroma-activity threshold, 14 odor-active compounds were identified across the four stages (i.e., compounds with ROAV ≥ 1 in at least one processing stage), of which eight were shared by at least two stages (Table 3).

The ROAV-based aroma profiles of the four stages are presented in Table 3. In raw grain (K1), vanillin served as the reference standard (ROAV = 100) and dominated the aroma profile, while 2-undecanone (ROAV = 6.46, orange/green) was a minor contributor. Germination (K2) introduced 2,3-butanediol (ROAV = 3.07, fruity/creamy), nonanal (ROAV = 2.08, citrus/orange peel), and butyl acetate (ROAV = 14.58, sweet/banana) as stage-specific markers. Roasting (K3) was characterized by a substantial increase in Maillard-derived compounds, including: 2,3,5-trimethylpyrazine (ROAV = 45.84, roasted/nutty) surged via Maillard reactions, accompanied by furfural (ROAV = 1.50, bread/almond), establishing the characteristic roasted aroma framework. In finished beer (K4), isoamyl alcohol (ROAV = 71.59, sweet/malty/banana) and 2-undecanone (ROAV = 65.37) became dominant, while yeast-derived esters—including phenethyl acetate (ROAV = 1.67, rose/honey) and hexadecanoic acid ethyl ester (ROAV = 5.08, waxy)—further enriched the sweet/floral dimension.

### 2.3. Non-Volatile Metabolites in Black Highland Barley Malt at Different Processing Stages

#### 2.3.1. Multivariate Statistical Analysis

**Figure 4:**
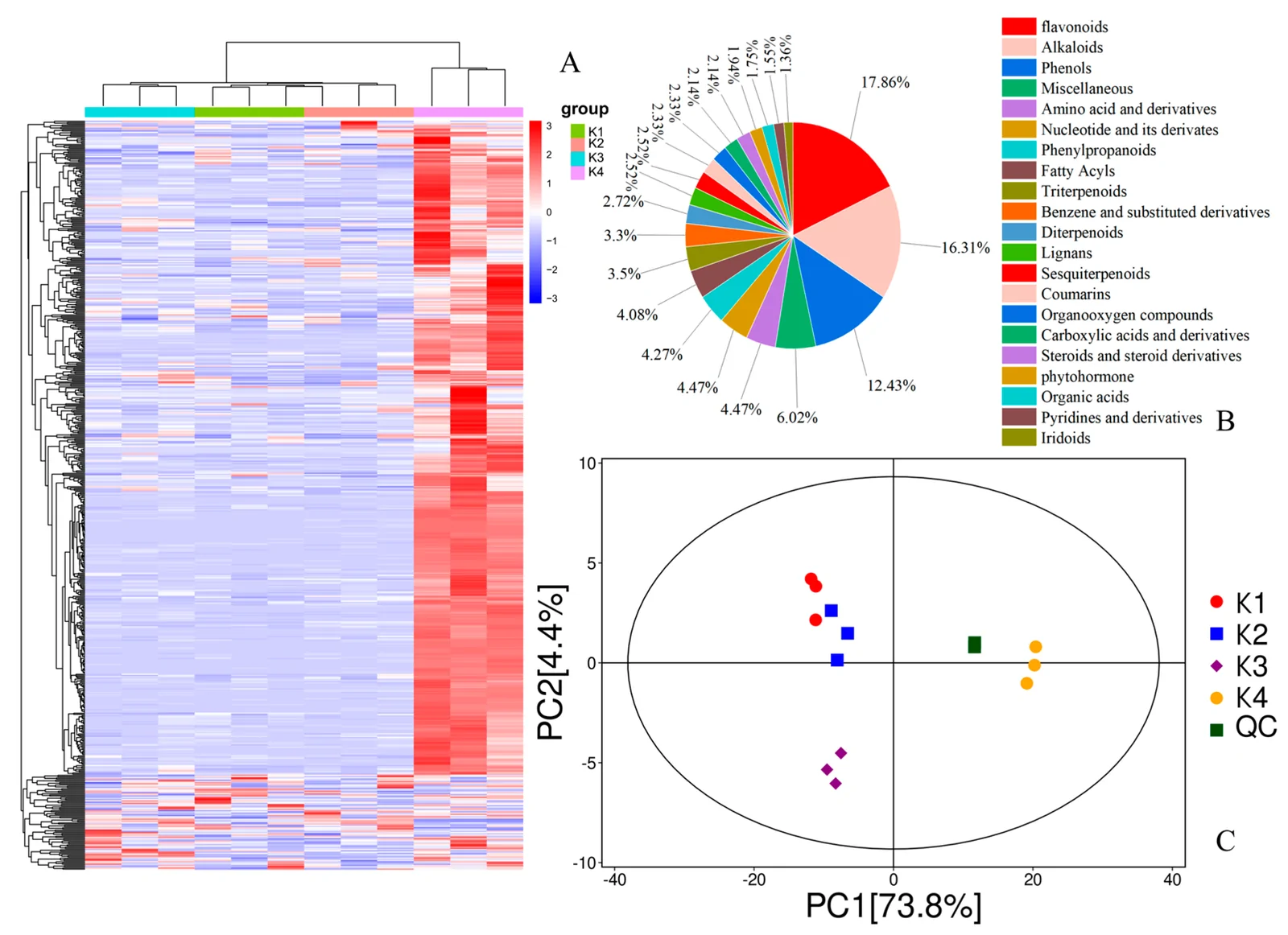
Multivariate statistical analysis of metabolites in black highland barley malt at different processing stages. (**A**) Clustering heatmap of metabolites (sample names are provided in the figure); (**B**) classification and composition of metabolites, Legend items are arranged from top to bottom in descending order of relative abundance; (**C**) principal component analysis of inter-group and intra-group metabolite distribution. K1–K4 as defined in Figure 1. Data are expressed as mean ± SD (n = 3).

Non-volatile metabolites also changed significantly with processing stages. Widely targeted metabolomics was applied to analyze raw material (K1), germinated malt (K2), roasted malt (K3), and black highland barley beer (K4), with QC sample chromatograms shown in Figure S2. We identified 615 metabolites in total (Figure 4A), which fell into 23 categories (Figure 4B). The major metabolite groups included flavonoids (99, 16.09%), alkaloids (84, 13.66%), and phenolic compounds and derivatives (67, 10.89%), whereas organic acids and derivatives (9, 1.46%) and pyridine derivatives (8, 1.30%) accounted for smaller proportions. PCA (Figure 4C) showed that QC samples clustered tightly, confirming the good reproducibility and reliability of the analytical method. PC1 (73.8%) and PC2 (4.4%) together explained 78.2% of the variance. Clear separation among the four groups was observed, with samples within each group clustering closely, indicating significant differences in metabolite composition across treatments. The K4 group exhibited more highly expressed metabolites compared with the other three groups, with a distinctly different expression pattern (Figure 4A). These results highlight that throughout the processing chain from malt preparation and roasting to beer fermentation, metabolites are continuously reshaped, with germination and roasting stages laying an important material foundation for metabolite enrichment and transformation during subsequent fermentation.

#### 2.3.2. Screening of Differential Metabolites

**Figure 5:**
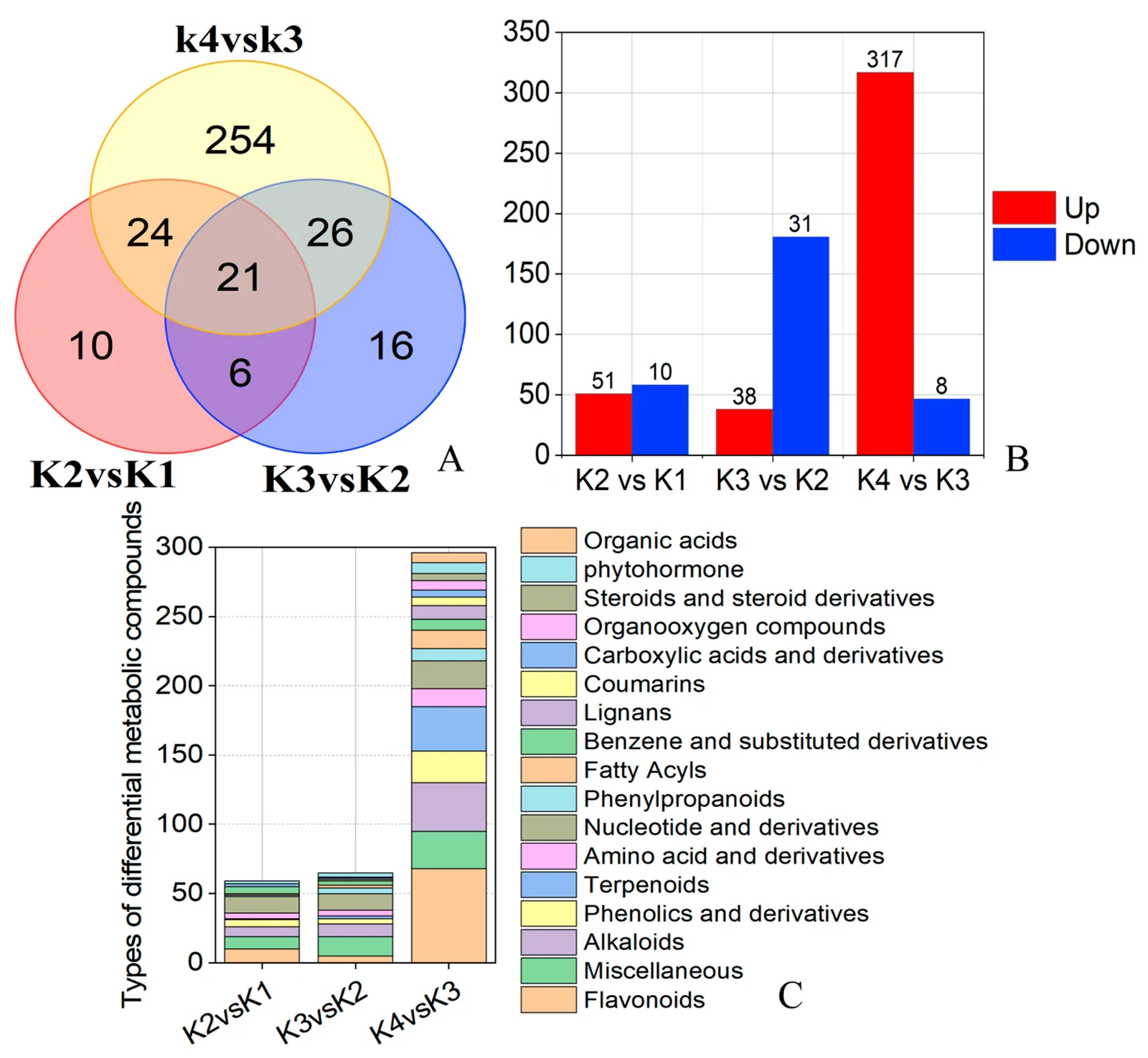
Statistical overview of differential metabolites in black highland barley malt at different processing stages. (**A**) Venn diagram showing the distribution of unique and shared differential metabolites across comparisons; (**B**) statistics of upregulated and downregulated differential metabolites; (**C**) classification of differential metabolites, Legend items correspond to the stacked color blocks from bottom to top. K1–K4 as defined in Figure 1. Data are expressed as mean ± SD (n = 3).

**Figure 6:**
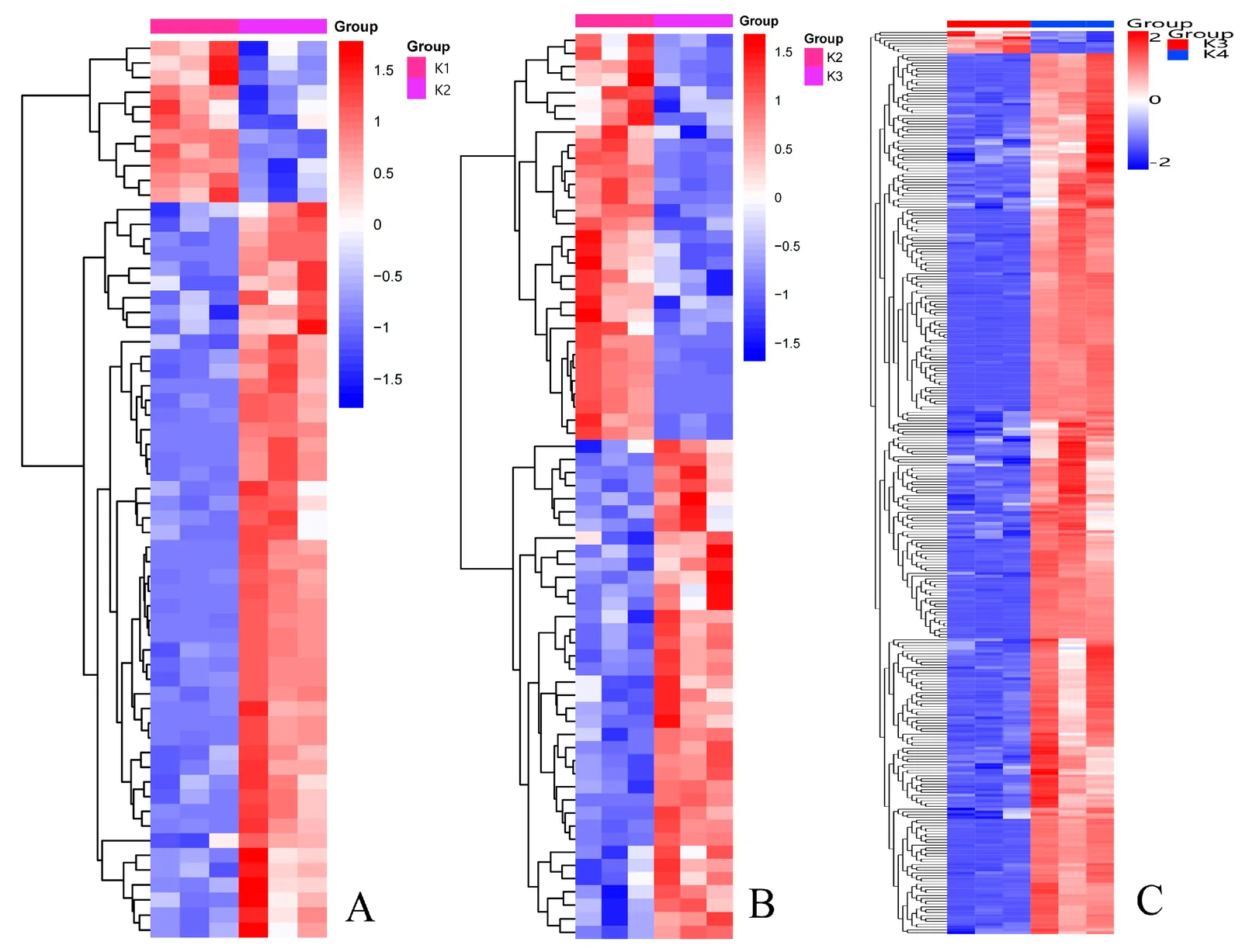
Heatmaps of differential metabolites in black highland barley malt at different processing stages. (**A**) K2 vs. K1, (**B**) K3 vs. K2, (**C**) K4 vs. K3 differential metabolite heatmaps. K1–K4 as defined in Figure 1. Data are expressed as mean ± SD (n = 3).

Under VIP > 1 and *p* < 0.05, 61 (K2 vs. K1), 69 (K3 vs. K2), and 325 (K4 vs. K3) differential non-volatile metabolites were identified, predominantly comprising nucleotides and derivatives, flavonoids, and alkaloids (Figure 5A,B). Among these, 10, 16, and 254 unique differential metabolites were observed in the three comparison groups, respectively, indicating that each processing stage induced distinct metabolic shifts, with fermentation exerting the most pronounced effect (Figure 5C). Twenty-one metabolites were consistently differential across all comparisons, including (+)-abscisic acid, 2-naphthol, 5′-deoxyadenosine, 5′-S-methyl-5′-thioadenosine, 5-thymidylic acid, adenosine, cellohexaose, cinnamyl cinnamate, 2′-deoxyguanosine, guanosine, guanosine 3′,5′-cyclic monophosphate, itaconic acid, N1-methyl-2-pyridone-5-carboxamide, N,N-dimethyl-1,4-phenylenediamine, nicotinamide, palmitoylethanolamide, riboflavine, thymidine, xanthosine, 2-phenylacetamide, and 3-hydroxy-3-methylpentane-1,5-dioic acid. As shown in Figure 6A–C, most of these metabolites exhibited marked accumulation after roasting and further enrichment in beer, with overall increases of approximately 17-fold (adenosine), 240-fold (5′-deoxyadenosine), 410-fold (2′-deoxyguanosine), 260-fold (cinnamyl cinnamate), 360-fold (guanosine), and 212-fold (riboflavin). In contrast, palmitoylethanolamide showed a continuous downward trend from raw material to finished beer.

Heatmap visualization (Figure 6A–C) revealed stage-specific metabolic patterns. During germination (K2 vs. K1), amino acid derivatives (e.g., 5-aminovaleric acid, pyrrole-2-carboxylic acid), phenylpropanoids (cinnamyl cinnamate), flavonoids (quercetin, diosmetin), and nucleotides (guanosine 3′,5′-cyclic monophosphate) were significantly upregulated, while certain alkaloids (isatin, piperidine) and abscisic acid decreased. During roasting (K3 vs. K2), L-ornithine, trans-caffeic acid, vanillin, epicatechin, and gentianine accumulated substantially, whereas most nucleotides (adenosine, thymidine) and some carboxylic acids declined. The brewing stage (K4 vs. K3) exhibited the most pronounced changes, with 317 metabolites upregulated and only 8 downregulated. Upregulated compounds included amino acid derivatives (L-ornithine, N-acetyl-L-phenylalanine), phenylpropanoids (cinnamyl cinnamate, ferulic acid, methyl cinnamate), phenolics (gentisic acid, phenol), and flavonoids (epicatechin, astragalin, rutin).

Of the 184 phenolic and flavonoid compounds identified via widely targeted metabolomics, the vast majority (168 compounds, 94.1%) exhibited higher abundances in finished beer (K4) relative to raw grain (K1), following a consistent multiphasic accumulation trajectory-a progressive decline during malting (K2) and roasting (K3), followed by a pronounced resurgence during fermentation (K4). This pattern was exemplified by nobiletin (K4/K1 = 262.5-fold), cinnamyl cinnamate (259.2-fold), and astragalin, among others (Figure S3).

#### 2.3.3. KEGG Pathway Enrichment for Differential Metabolites

**Figure 7:**
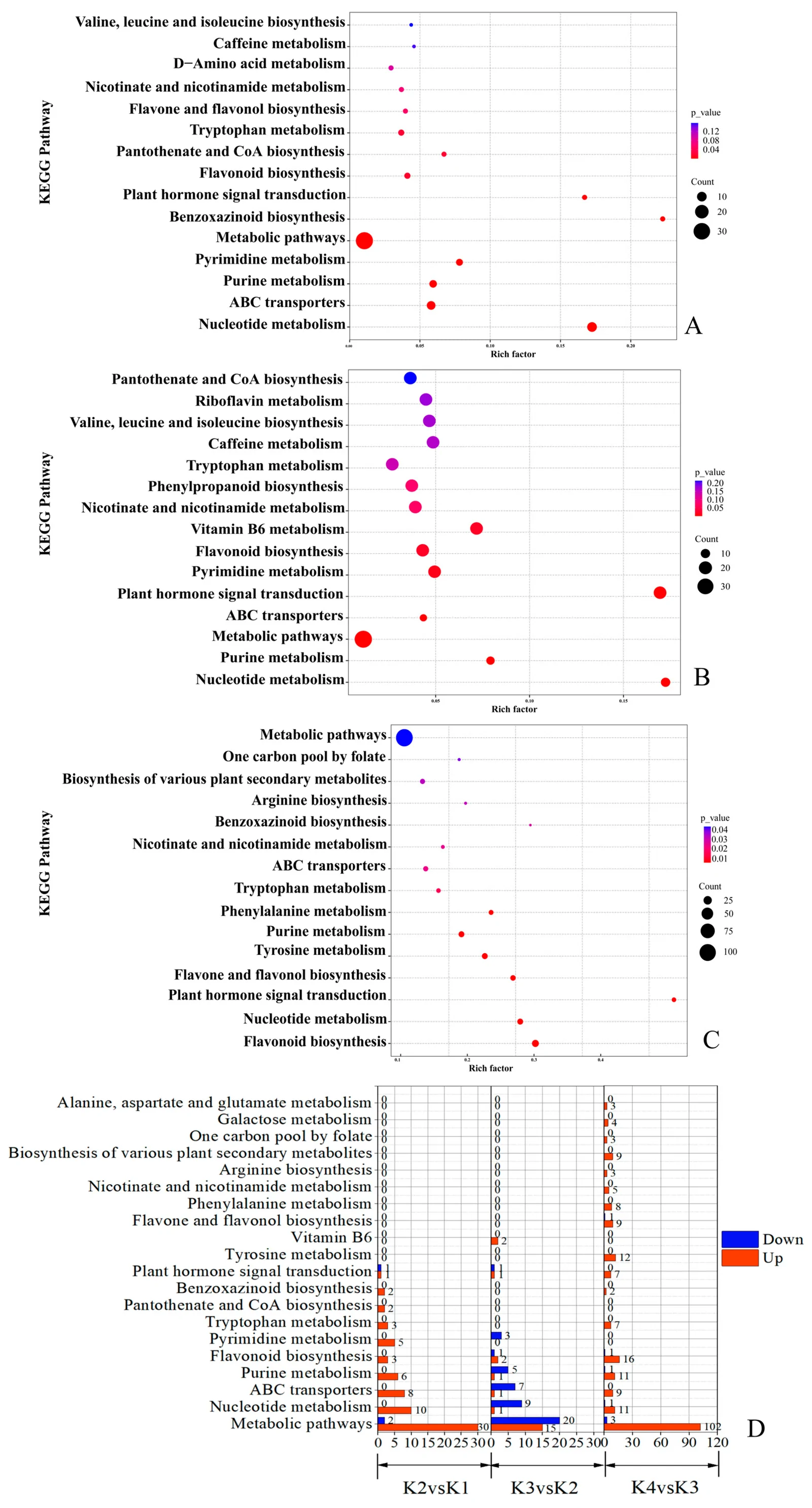
KEGG enrichment of differential metabolites in black highland barley malt at different processing stages. (**A**) K2 vs. K1; (**B**) K3 vs. K2; (**C**) K4 vs. K3; (**D**) statistics of upregulated and downregulated differential metabolites in KEGG pathways. K1–K4 as defined in Figure 1. Data are expressed as mean ± SD (n = 3).

KEGG pathway enrichment analysis (Figure 7A–D) revealed distinct stage-specific metabolic reprogramming across the three processing stages.

For germination (K2 vs. K1), differential metabolites were significantly enriched in nucleotide metabolism, purine metabolism, and ABC transporters, with 30, 10, and 6 upregulated compounds, respectively. Thymidine and uracil accumulated during germination.

For roasting (K3 vs. K2), nucleotide metabolism became predominantly downregulated (9 down vs. 1 up), and ABC transporters and purine metabolism also showed significant suppression. Vitamin B6 metabolism was specifically upregulated.

For beer fermentation (K4 vs. K3), upregulated compounds in metabolic pathways and flavonoid biosynthesis reached 102 and 16, respectively. Tyrosine metabolism and phenylalanine metabolism emerged as uniquely significant at this stage, accompanied by substantial accumulation of phenolic compounds such as phenol, salicylic acid, and ferulic acid.

### 2.4. Correlation Analysis

**Figure 8:**
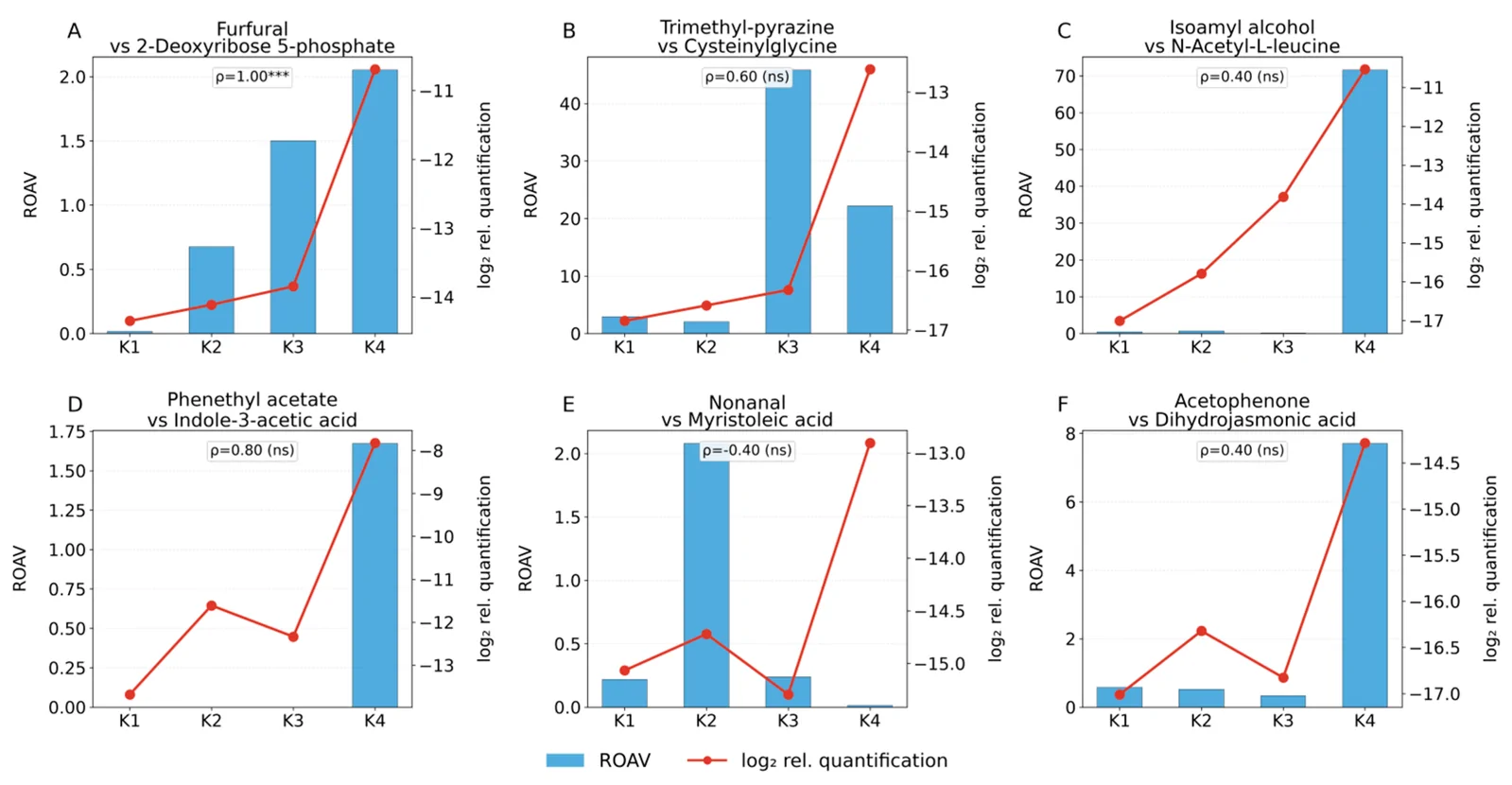
Stage-level Spearman correlations between aroma-active compounds (ROAV ≥ 1) and their potential non-volatile precursors across four processing stages (K1–K4). Bars represent ROAVs (left y-axis); red lines with markers indicate log_2_-transformed precursor intensities (right y-axis). Spearman rank correlation coefficients (ρ) and *p*-values are shown in each panel. ns, not significant (*p* ≥ 0.05); ***, *p* < 0.001. Mean values of three biological replicates were used per stage (n = 4 stages). K1–K4 as defined in Figure 1.

To reveal the metabolic basis of aroma formation, stage-level Spearman correlation analysis was performed between the ROAVs of aroma-active volatiles (ROAV ≥ 1) and the log_2_-transformed intensity of their proposed biochemical precursors across the four processing stages (K1–K4, n = 4 stage means). Pair selection was based on known biochemical pathways rather than correlation strength alone, covering Maillard reactions, Ehrlich pathway, esterification, lipid peroxidation, and phenolic oxidation (Figure 8).

Among Maillard-derived aromas, furfural showed a strong positive correlation with the pentose phosphate pathway intermediate 2-deoxyribose-5-phosphate (ρ = 1.00, *p* < 0.001, Figure 8A). Trimethylpyrazine exhibited a non-significant positive trend with cysteinylglycine (ρ = 0.60, Figure 8B). For the Ehrlich pathway, isoamyl alcohol trended positively with the leucine catabolism intermediate N-acetyl-L-leucine (ρ = 0.40, Figure 8C). In yeast esterification, phenethyl acetate showed a non-significant positive trend with indole-3-acetic acid (ρ = 0.80, Figure 8D). For lipid-derived volatiles, nonanal exhibited a non-significant negative trend with its unsaturated fatty acid precursor myristoleic acid (ρ = −0.40, Figure 8E). For phenolic-related volatiles, acetophenone showed a non-significant positive trend with dihydrojasmonic acid (ρ = 0.40, Figure 8F).

Among all screened pairs, only the furfural–pentose phosphate relationship reached statistical significance. The remaining non-significant trends highlight the complexity of multi-step metabolic transformations and the limitation of stage-level correlation (n = 4) for detecting weaker associations. Future studies with higher temporal resolution are warranted to further resolve these precursor–product relationships.

## 3. Discussion

### 3.1. Malt Modification and Roasting Characteristics

The steeping degree determined in this study was 47.5% ± 0.6%, which falls within the optimal range for brewing malts (43–48%), confirming that the steeping process was appropriately controlled. Notably, the Kolbach index (54%) and diastatic power (108 °WK) were higher than typical values for conventional pale malts (Kolbach: 38–45%; diastatic power: 60–80 °WK). This apparent discrepancy—normal steeping degree yet elevated modification indices—suggests that black highland barley possesses intrinsically higher enzymatic activity and protein solubility compared with conventional malting barley cultivars. This characteristic is likely attributable to the smaller kernel size of the Nangqên black highland barley used in this study, which provides a larger specific surface area and facilitates more efficient water uptake and enzymatic modification during germination, as well as its distinct protein composition and germination vigor. Therefore, the elevated Kolbach index and diastatic power reflect the inherent malting characteristics of this black highland barley variety rather than process-induced over-modification. The present malting protocol was optimized for metabolite profiling rather than for commercial-scale pale malt production, and the findings should be interpreted as characterizing the metabolic trajectories under the specific conditions employed. Future studies should systematically optimize steeping and germination regimes for black highland barley to achieve a balanced modification level suitable for various beer styles.

The roasting temperature (100–110 °C) employed in this study was selected to produce a specialty malt with pronounced roasted and caramel-like aroma characteristics while preserving sufficient enzymatic activity for subsequent fermentation. This temperature regime promotes Maillard reactions and caramelization, generating the characteristic pyrazines and furans identified in this study (e.g., 2,3,5-trimethylpyrazine and furfural). Consequently, the finished beer exhibited a color of approximately 16 EBC at 8.8 °P, which places it outside the pale lager category. Therefore, the findings of this study are most relevant to dark specialty beers brewed with roasted colored grains, and extrapolation to conventional pale beers should be made with caution. Future studies should systematically compare the metabolic and flavor profiles of pale and roasted malts derived from the same black highland barley genotype to delineate the specific contributions of roasting temperature to final beer quality.

### 3.2. Volatile and Non-Volatile Metabolic Dynamics During Processing

The volatile flavor profiles of black highland barley products changed substantially across the four processing stages. The increase in aldehydes such as nonanal and benzeneacetaldehyde during germination imparts pleasant floral and fruity notes, which is consistent with previous reports in germinated millet and soybean [28,29]. The reduction in ester content during germination may be related to their consumption as respiratory substrates [30]. The substantial accumulation of 2,3,5-trimethylpyrazine and furfural during roasting is consistent with Maillard reaction chemistry [31], imparting intense roasted, nutty, and sweet-caramel aromas to the malt. The depletion of limonene during roasting is likely due to thermal degradation or volatilization. In finished beer, esters became the predominant class (40.95%), which is attributable to yeast metabolism during fermentation, as yeast produces a wide range of esters through the condensation of alcohols with acyl-CoA [32]. The decrease in 1-octen-3-ol during fermentation is notable because at high concentrations it imparts undesirable mushroom-like and earthy off-flavors [33]; its substantial reduction likely contributes to a cleaner, more refined flavor profile. The concomitant decline of harsh aldehydes such as decanal and nonanal further supports the flavor-improving effect of fermentation [34]. The ROAV-based analysis further identified 14 key aroma-active compounds, among which 2,3,5-trimethylpyrazine (ROAV = 45.84) and furfural (ROAV = 1.50) were the dominant contributors to the roasted aroma in K3, while isoamyl alcohol (ROAV = 71.59) and 2-undecanone (ROAV = 65.37) emerged as the principal aroma contributors in K4. The clear separation between K1/K2 and K3/K4 in the OPLS-DA score plot reflects the distinct volatile profiles acquired during roasting and fermentation, consistent with the pronounced volatile profile transformation driven by roasting and fermentation of black highland barley products.

The non-volatile metabolomics analysis revealed extensive metabolic remodeling during processing, particularly during fermentation. The observed changes in non-volatile metabolites during processing may contribute to aroma formation, as several of these compounds serve as flavor precursors. The accumulation of L-ornithine during roasting and its further increase in beer is consistent with carboxypeptidase activation during malting, which releases free amino acids from protein degradation [35]. The phenolic and flavonoid accumulation pattern observed in black highland barley beer is consistent with previous reports on highland barley beers, which have been shown to possess higher total flavonoid and polyphenol contents compared with beers brewed from other cereals, thereby exhibiting enhanced antioxidant capacity [36,37,38]. The most pronounced metabolic remodeling occurred during fermentation, with 317 metabolites upregulated and only 8 downregulated, suggesting that fermentation serves as the decisive metabolic turning point that simultaneously shapes the flavor profile and nutritional quality of black highland barley beer.

KEGG pathway enrichment analysis further elucidated the stage-specific metabolic reprogramming. The accumulation of thymidine and uracil during germination reflects enhanced DNA/RNA synthesis and energy metabolism during seed germination, consistent with the activation of endogenous enzyme systems that degrade starch and proteins to supply energy and precursors for subsequent growth [39]. The upregulation of vitamin B6 metabolism during roasting suggests that thermal effects regulate vitamin metabolism, a finding closely linked to Maillard reaction precursor formation and malt flavor development [39,40]. The emergence of tyrosine and phenylalanine metabolism during fermentation, accompanied by the accumulation of phenolic compounds such as phenol, salicylic acid, and ferulic acid, indicates that yeast fermentation actively transforms malt precursors to enrich both flavor and functional characteristics of the final product.

The cross-omics correlation analysis provided preliminary insights into precursor–product relationships linking aroma-active volatiles to their proposed biochemical precursors. The strong correlation between furfural and its pentose phosphate pathway intermediate 2-deoxyribose-5-phosphate (ρ = 1.00, *p* < 0.001) underscores the tight coupling between thermal pentose phosphate degradation and Maillard-derived caramel aroma generation during roasting. The positive trend between isoamyl alcohol and N-acetyl-L-leucine is consistent with the Ehrlich pathway, in which leucine is catabolized to isoamyl alcohol. The positive trend between phenethyl acetate and indole-3-acetic acid is consistent with the concurrent accumulation of auxin-related metabolites and ester biosynthesis during fermentation. The remaining non-significant trends, while mechanistically plausible, highlight the complexity of multi-step metabolic transformations and the limitation of stage-level correlation (n = 4) for detecting weaker associations.

Collectively, these pathway-level dynamics demonstrate that germination primarily activates energy- and precursor-supplying pathways; roasting redirects metabolism toward flavor precursor formation through thermal-driven transformations; and fermentation further amplifies phenolic metabolism and secondary biosynthesis, ultimately shaping the distinctive quality of black highland barley beer. These findings underscore the importance of optimizing germination conditions, roasting regimes, and brewing parameters for targeted quality improvement.

### 3.3. Comparison with Other Brewing Cereals and Future Perspectives

The unique phenolic and flavonoid accumulation pattern observed in black highland barley beer warrants comparison with other brewing cereals used as adjuncts or alternative grain sources. Recent studies have demonstrated that substituting barley malt with milled rice in non-alcoholic beer brewing can modulate sensory and chemical characteristics by reducing aldehyde levels and diminishing undesirable “worry” flavor, although rice lacks the phenolic richness of pigmented grains such as black highland barley [41]. Similarly, the use of koji in combination with barley malt has been shown to influence ester formation and amino acid profiles during fermentation, highlighting the importance of grain composition and enzymatic potential in determining final beer flavor [42]. Interestingly, different rice cultivars exhibit marked variations in malting suitability and brewing quality, with some varieties producing beers with distinct flavor characteristics comparable to those of barley malt [43,44]. These findings support the notion that cereal genotype significantly influences both the metabolic and sensory outcomes of brewing. Compared with rice, which is relatively neutral in flavor, black highland barley contributes not only fermentable sugars but also a rich pool of phenolic compounds and flavonoids that are retained and even enriched during fermentation, as demonstrated in this study. This metabolic resilience distinguishes black highland barley from more conventional brewing cereals and underscores its potential as a functional ingredient for specialty beer production. The distinct phenolic trajectories observed in the present study therefore add to the growing body of evidence that grain selection is a critical determinant of both the flavor complexity and nutritional value of the final beer.

Despite the relevance of comparing the four processing stages, it should be acknowledged that the specific malting and roasting conditions employed in this study—particularly the relatively high Kolbach index (54%) and the use of roasting at 100–110 °C—limit the generalizability of the beer-related findings. The resulting beer (approximately 16 EBC at 8.8 °P) is characteristic of a dark specialty style rather than a conventional pale lager, and extrapolation to other beer styles or malting protocols should be made with caution. In addition, hop-derived compounds could not be fully distinguished from malt-derived metabolites in the final beer, as hops were added during the boiling stage to produce a representative product. Therefore, the beer metabolomics and flavoromics data reflect the combined contributions of barley malt, hops, and yeast metabolism. Future studies should investigate the effects of varying malting and roasting parameters, and consider hop-free controls or stable isotope labeling to further distinguish hop-derived metabolites from barley-derived ones.

Overall, the findings of this study provide a comprehensive view of the metabolic and flavor changes occurring during black highland barley beer production. The integration of flavoromics and widely targeted metabolomics revealed that each processing stage—germination, roasting, and fermentation—makes distinct contributions to the final product, with fermentation serving as the decisive step for both flavor development and phenolic enrichment. These results highlight the potential of black highland barley as a premium raw material for specialty beer production and offer a scientific basis for optimizing processing parameters to achieve desired flavor and nutritional profiles.

## 4. Materials and Methods

### 4.1. Materials and Equipment

The black highland barley (*Hordeum vulgare* L.) germplasm resources from Nangqian were provided by the Crop Research Institute, Qinghai Academy of Agricultural and Forestry Sciences (Xining, China). The identity and varietal purity of the black highland barley were verified by the provider institute through morphological examination of key diagnostic traits, including black-purple pericarp color, hull-less grain type, grain shape, and spike density, following the guidelines for distinctness, uniformity, and stability (DUS) testing of barley (GB/T 19557.31-2018) [45]. Upon harvest (25 August 2023), the grains were cleaned by winnowing combined with manual selection to remove broken kernels and foreign matter. The cleaned grains were homogenized by coning and quartering to ensure representative sampling and stored in woven plastic bags in a warehouse with an annual mean temperature ranging from 4 to 10 °C until processing. The initial moisture content of the raw grain was determined by oven-drying at 105 °C to constant weight and found to be 10.56% ± 0.15% (w.b.). The barley was sown on 1 May 2023, and harvested on 25 August 2023. The cultivation site was located in Bai’zha Township, Nangqian County (96°51′ N, 32°07′ E; altitude: 4070 m).

Analytical-grade ethanol, sodium chloride, n-hexane, methanol, and acetonitrile were obtained from standard commercial suppliers (Aladdin (Shanghai, China), Sinopharm (Shanghai, China), Yonghua Chemical (Suzhou, China), CNW Technologies, (Shanghai, China)). Formic acid, saturated alkane standards, and n-hexanol-d13 alcohol were purchased from Sigma-Aldrich (St. Louis, MO, USA) and C/D/N Isotopes (Pointe-Claire, QC, Canada). Ultrapure water was used throughout the experiments (Table 4).

### 4.2. Experimental Methods

#### 4.2.1. Black Highland Barley Malt and Beer Preparation

Steeping: The total steeping time for black highland barley (K1) was 50 h, consisting of three wet steeping phases and two dry steeping (draining) phases. The process began by placing the barley into a 170 L tank for an initial wet steep at 12 °C for 4 h. This step was followed by a 20 h dry steeping period at 17 °C.

Germination: After steeping, the barley was evenly spread in a germination trough and incubated at 20 ± 1 °C in a constant-temperature chamber. Germination was continued until more than 80% of the grains had sprouted, with sprout length reaching 70–80% of the grain height, yielding germinated malt (K2).

Roasting and de-rooting: After germination, the grains were drained and dried in a roasting machine at 100–110 °C to reduce moisture content and develop roasted aroma characteristics through Maillard reactions. Subsequently, the rootlets were removed to obtain roasted malt (K3), which was then packed and stored in a dry, well-ventilated warehouse.

Black Highland Barley Beer (K4) Fermentation: Black highland barley beer was brewed using black highland barley malt as the primary raw material, through processes including crushing, mashing, filtration, boiling, fermentation, and maturation. After crushing to a moderate coarse grind, with the pericarp largely intact and starch granules ruptured, the black highland barley malt was mixed with purified water, with the proportions being 1.0 g of malt per 4.5 mL of water. The stepwise mashing consisted of four consecutive phases: enzyme extraction (35 °C, 15 min), protein rest (50 °C, 50 min), saccharification (65 °C, 60 min), and enzyme inactivation (78 °C, 10 min). After static settling, the mash was filtered using three-cycle reflux filtration and sparged twice with 78 °C hot water to adjust the wort to the target gravity. The filtered wort was boiled for 60 min, with Saaz hops added in two portions (total 2.5 g/L; 50% at the start of boiling and the remaining 50% at 10 min before the end). After boiling, the wort was cooled to 20 °C via a coil cooler, and activated dry yeast (Angel CS31 (Saccharomyces cerevisiae), Angel Yeast Co., Ltd., Yichang, China) (1 g/L, activated in sterile water at 20 °C for 20 min) was pitched. The target original gravity of the wort was adjusted to 10 °P by sparging. Primary fermentation was conducted at 12 °C for approximately 14 days until the gravity dropped to 5 °P. After adding 2.2 g/L sucrose, the beer was bottled and subjected to secondary fermentation at the same temperature for 14 days, followed by cold storage at 4 °C for 3 days to obtain the finished black highland barley craft beer.

#### 4.2.2. Flavor Compound Determination

Volatile extraction and GC×GC-TOF MS analysis were performed by NovoMagic Co., Ltd. (Suzhou, China).

Sample preparation: A 1 mL sample was pipetted into a 20 mL headspace vial, mixed with 2 mL saturated aqueous sodium chloride solution (approximately 360 g/L at room temperature) and 10 μL internal standard (n-hexanol-d13, 10 mg/L, 98.5% purity, C/D/N Isotopes, Montreal, QC, Canada), then incubated at 80 °C for 10 min. Peak areas were normalized against the internal standard (A_i/A_IS) for semi-quantitative determination; the IS-normalized peak area ratio was used to calculate relative concentrations for subsequent ROAV evaluation. A SPME fiber (DVB/CAR/PDMS, 50/30 μm × 1 cm, StableFlex, Supelco 57329-U, Bellefonte, PA, USA) was conditioned at 270 °C (10 min), inserted into the vial for headspace adsorption (80 °C, 30 min), desorbed in the GC inlet (250 °C, 5 min), and cleaned at 270 °C (10 min). System validation used 10 μL saturated alkanes (1 mg/L) under the same conditions [46,47].

GC×GC-TOF MS: LECO Pegasus 4D (Agilent 8890A GC, dual-stage modulator, TOFMS). Columns: DB Heavy Wax (30 m × 0.25 mm, 0.25 μm) as 1D, Rxi-5Sil MS (2.0 m × 0.15 mm, 0.15 μm) as 2D. Carrier gas: He (>99.999%), constant 1.0 mL/min. Inlet: 250 °C, splitless. Oven: 1D from 40 °C (3 min) to 250 °C at 5 °C/min (5 min); 2D 5 °C above 1D. Modulation: period 4.0 s, 15 °C above 2D. TOFMS: EI 70 eV; source/transfer line 250 °C; *m*/*z* 35–550 at 200 spectra/s; detector 2008 V.

#### 4.2.3. Metabolite Determination

Non-volatile metabolite extraction and UHPLC-MS analysis were performed by Shanghai Biotree Biotech Co., Ltd. (Shanghai, China).

Sample preparation: About 50 mg of each lyophilized sample was placed into a 1.5 mL polypropylene tube, then 700 μL of ice-cold (−40 °C) extraction solvent (methanol: water = 3: 1, *v*/*v*, containing internal standards) was added. The mixture was vortexed (30 s), homogenized in an ice bath (40 Hz, 4 min), and sonicated (5 min); this cycle was repeated three times. After overnight extraction with shaking at 4 °C, the samples were centrifuged (12,000 rpm, 15 min, 4 °C; RCF ≈ 13,800× *g*), and the supernatants were filtered through 0.22 μm nylon membranes.

UHPLC-MS: A Sciex EXIONLC™ system with a Waters ACQUITY UPLC HSS T3 column (1.8 μm, 2.1 × 100 mm) was used. Mobile phase: A = 0.1% formic acid in water, B = acetonitrile. Conditions: column 40 °C, autosampler 4 °C, injection 2 μL, flow 0.4 mL/min. Gradient: 0–0.5 min (98% A), 0.5–10 min (98% A), 10–11 min (50% A), 11–13 min (5% A), 13–13.1 min (98% A), 13.1–15 min (98% A).

MS detection: Sciex QTrap 6500^+^. Ion spray voltage: +5500 V (positive), −4500 V (negative); curtain gas: 35 psi; temperature: 400 °C; GS1/GS2: 60 psi; declustering potential: ±100 V.

#### 4.2.4. Determination of Physicochemical and Nutritional Parameters

The nutritional composition of raw grain and malt was determined as follows. Protein was measured by the Kjeldahl method using a Gerhardt Kjeldahl system (Gerhardt, Königswinter, Germany) according to the Chinese national standard GB 5009.5 [48]. Fat was determined by Soxhlet extraction using a Gerhardt Soxhlet extractor (Gerhardt, Königswinter, Germany) according to GB 5009.6 [49]. Crude fiber was determined using a Gerhardt fiber analyzer (Gerhardt, Königswinter, Germany) according to GB/T 5009.10 [50]. Ash was determined according to GB 5009.4 [51]. Amino acids were analyzed by acid hydrolysis according to GB 5009.124 [52]. Total starch, amylose, and β-glucan were determined using Megazyme assay kits (K-TSTA-100A for total starch, K-AMYL for amylose/amylopectin, and K-BGLU for β-glucan; Megazyme International, Wicklow, Ireland) following the manufacturers’ protocols. Total and soluble dietary fiber were measured according to the method of Dang et al. [53].

Wort quality parameters (pH, color, extract content, α-amino nitrogen, saccharification power, and Kolbach index) were analyzed according to QB/T 1686-2008 [54]. Methods of Analysis of Malt for Brewing and the associated standard protocols referenced therein. pH was measured using a pH meter according to GB/T 4928-2008 [55]. Color of wort was determined spectrophotometrically at 430 nm using a Purkinje TU-1810 spectrophotometer (Purkinje General Instrument Co., Ltd., Beijing, China) and expressed in EBC units according to QB/T 1686-2008 [54]. The Kolbach index was calculated as the ratio of soluble nitrogen to total nitrogen according to the Institute of Brewing (IoB) recommended procedures. Extract content, α-amino nitrogen, and saccharification power were determined according to QB/T 1686-2008 [54].

Beer physicochemical indices were analyzed according to the Chinese national standard GB/T 4928-2008 [55] (Analytical methods of beer). Alcohol content was determined by distillation. Original extract and apparent extract were determined using a glass hydrometer (range: 0.8–0.9, division: 0.001. Hengshui Yaohua Instruments Factory, Hengshui, China) at 20 °C. Alcohol content was calculated from the difference between original and apparent extract according to GB/T 4928-2008 [55]. Color was determined spectrophotometrically at 430 nm using a Purkinje TU-1810 spectrophotometer (Purkinje General Instrument Co., Ltd., Beijing, China) and expressed in EBC units. Total acidity was measured by titration according to the Chinese national standard GB 12456-2021 [56] (National food safety standard—Determination of total acid in foods). Reducing sugar was determined according to the Chinese national standard GB 5009.7 [57] (National food safety standard—Determination of reducing sugar in foods). Soluble solids were measured using a handheld refractometer at 20 °C according to the Chinese national standard GB/T 12143-2008 [58] (General analytical methods of beverage—Determination of soluble solids—Refractometric method). Fermentation degree was calculated as the ratio of apparent extract to original extract according to GB/T 4928-2008 [55].

Total phenolics, total flavonoids, and antioxidant activities were determined as follows. Total phenolics were measured by the Folin–Ciocalteu method [59], using gallic acid as the standard, and results were expressed as mg gallic acid equivalents per liter (mg GAE/L). Total flavonoids were measured by the aluminum chloride colorimetric method [60], using rutin as the standard, and results were expressed as mg rutin equivalents per liter (mg RE/L). DPPH radical scavenging activity and ABTS radical scavenging activity were determined according to [61,62].

### 4.3. Statistical Analysis

All experiments were performed in triplicate, and data are expressed as mean ± standard deviation (SD). For physicochemical parameters (Table 1 and Table 2), pairwise comparisons between stages where both parameters were determined were assessed by independent-samples *t*-tests (K1 vs. K2 for Table 1; K3 vs. K4 for Table 2), with significance set at *p* < 0.05. Different superscript letters indicate significant differences. Parameters determined at only a single stage are presented as descriptive statistics (mean ± SD) without significance letters. For volatile metabolites, differentially abundant compounds across the four stages were identified by orthogonal partial least-squares discriminant analysis (OPLS-DA, SIMCA-P v13.0, Umetrics, Umeå, Sweden) combined with one-way ANOVA (*p* < 0.05) and Benjamini–Hochberg false discovery rate (FDR) correction; the variable importance in projection (VIP) threshold was set at ≥1.0. For non-volatile metabolites, one-way ANOVA with Benjamini–Hochberg FDR correction was applied. Volcano plots were generated at |log_2_ fold change| ≥ 1 and *p* < 0.05. MRM data were processed using SCIEX Analyst Work Station (version 1.6.3), and raw MS files (.wiff) were converted to TXT format using MSconverter (version 3.0.22290). PCA and OPLS-DA were conducted using SIMCA-P v13.0 (Umetrics, Umeå, Sweden). Compound identities were retrieved from FlavorDB (https://cosylab.iiitd.edu.in/flavordb/, accessed on 20 July 2026)), and pathway analysis from KEGG (http://www.kegg.jp, accessed on 20 July 2026)). Figures were generated using Origin 2021 (OriginLab, Northampton, MA, USA) and the lims2 platform (https://www.lims2.com/, accessed on 20 July 2026)).

Volatile aroma contributions were assessed via the relative odor activity value (ROAV), calculated as ROAV_i_ = (C_i/OT_i)/(C_ref/OT_ref) × 100, where C_i is the IS-normalized relative concentration of compound i, OT_i is its odor threshold retrieved from FlavorDB (https://cosylab.iiitd.edu.in/flavordb/, accessed on 20 July 2026)), C_ref and OT_ref are the corresponding values for the reference compound, and compounds with ROAV ≥ 1 are defined as aroma-active. Vanillin was selected as the reference compound (ROAV = 100) because it exhibited the maximum OAV (C/OT) among all 77 quantified volatiles across all four processing stages (K1–K4), making it a suitable internal reference for calculating proportional aroma contributions.

For the 14 aroma-active compounds identified with ROAV ≥ 1, semi-quantitative estimation was performed by one-point calibration relative to the internal standard (n-hexanol-d13). The relative concentration of each compound was calculated as the peak area ratio of the compound to that of the internal standard, and the estimated values are provided in Table S1.

For non-volatile metabolomics, 1203 raw features were initially detected by UHPLC-QTRAP-MS; following annotation, 615 metabolites with confirmed identities were retained (unannotated features and isotopic internal standards excluded), and mean peak areas of three replicates per stage (K1–K4) were log_2_-transformed. Stage-level Spearman rank correlations (n = 4 stage means, corresponding to K1–K4) were performed between the log_2_-transformed intensities of the 615 non-volatile metabolites and the ROAVs of volatile compounds, as well as between the ROAVs of the 14 aroma-active volatiles (ROAV ≥ 1) and the log_2_-transformed intensities of their pathway-matched precursors. Correlation coefficients (ρ) and Bonferroni-adjusted *p* values are reported, with *p* < 0.05 considered statistically significant.

## 5. Conclusions

This study mapped the dynamic changes in volatile and non-volatile metabolites across the entire processing chain of black highland barley beer, leading to three main findings. First, each processing stage makes distinct contributions to the final flavor profile. Germination activates enzyme systems and elevates aldehydes. Roasting generates Maillard reaction products, such as 2,3,5-trimethylpyrazine and furfural, which establish the roasted aroma framework. Fermentation drives ester biosynthesis and phenolic enrichment. Second, fermentation serves as the decisive metabolic turning point. A total of 94.1% of phenolic and flavonoid compounds (168 of 184) decreased after roasting and then increased substantially during fermentation, resulting in a finished beer with 250.45 mg GAE/L total phenolics, 97.91 mg RE/L total flavonoids, and strong in vitro antioxidant capacity (ABTS: 87.41%; DPPH: 92.68%), indicating that black highland barley possesses unique metabolic resilience during brewing. Third, cross-omics correlation identified a strong linkage between furfural and its pentose phosphate precursor (ρ = 1.00, *p* < 0.001), providing mechanistic insight into the thermal generation of roasted aroma compounds. Collectively, these findings provide a metabolomic and flavoromic basis for optimizing malting parameters, roasting regimes, and fermentation protocols, and support the use of black highland barley as a premium ingredient for producing beers with enhanced flavor complexity and nutritional value.

Limitations and future perspectives: Several limitations of this study should be acknowledged. The correlation analysis between volatile and non-volatile metabolites was conducted at the stage level (n = 4 stages) due to the nature of the processing chain design; although this approach captured major metabolic transitions, future studies employing higher temporal resolution would enable more robust precursor–product relationship inference. The antioxidant capacity of the finished beer was assessed solely by in vitro chemical assays, and future research should incorporate cellular antioxidant models and human bioavailability studies to better evaluate the physiological relevance of the phenolic compounds retained in black highland barley beer. While we identified numerous stage-specific metabolic shifts, the underlying enzymatic mechanisms—particularly those governing the unique phenolic accumulation during fermentation—require further investigation through transcriptomic or proteomic approaches. It should also be noted that hop-derived compounds could not be fully distinguished from malt-derived metabolites in the final beer, and future studies should consider hop-free controls or stable isotope labeling to further separate their respective contributions. In addition, the malting and roasting protocols used in this study were optimized for metabolite profiling rather than for commercial-scale production, and systematic optimization of processing parameters is needed to achieve a balanced modification level suitable for various beer styles. Sensory evaluation should also be conducted to validate the perceived flavor impact of the key aroma-active compounds identified by ROAV analysis, and absolute quantification using authentic standards and full calibration curves would further strengthen the characterization of the most sensorially relevant volatiles.

## Figures and Tables

**Table 1:** Basic nutritional composition of raw grain and malted black highland barley.

Parameter	Raw Grain (K1)	Malt (K2)
Steeping degree (%)	47.5 ± 0.6	-
Protein (%)	9.22 ± 0.74 ^a^	12.43 ± 0.04 ^b^
Total starch (%)	58.76 ± 1.15	55.71 ± 1.65
Amylose (%)	25.36 ± 0.03 ^a^	24.37 ± 0.14 ^b^
β-glucan (%)	3.66 ± 0.13 ^a^	2.56 ± 0.20 ^b^
Fat (%)	1.91 ± 0.59	1.76 ± 0.37
Total dietary fiber (%)	18.17 ± 1.00 ^a^	16.09 ± 0.11 ^b^
Soluble dietary fiber (%)	2.69 ± 0.06 ^a^	8.73 ± 0.07 ^b^
Crude fiber (%)	3.43 ± 0.46	4.09 ± 0.07
Ash (%)	1.56 ± 0.04 ^a^	2.10 ± 0.01 ^b^
Total amino acids (mg·g^−1^)	-	8.105
Essential amino acids (mg·g^−1^)	-	2.76
Kolbach index (%)	-	53.59

Note: Data are expressed as mean ± SD (n = 3). Different superscript letters in the same row indicate significant differences between K1 and K2 (independent-samples *t*-test, *p* < 0.05). Parameters marked as “-” were not determined for that stage and were not included in statistical comparisons. ns (no superscript letters) indicates no significant difference (*p* ≥ 0.05).

**Table 2:** Brewing quality parameters of roasted black highland barley malt and finished beer quality.

Parameter	Roasted Malt (K3)	Finished Beer (K4)
pH	6.18 ± 0.03 ^a^	4.30 ± 0.07 ^b^
Extract (%)	72.87 ± 0.50	-
Color (EBC)	14.26 ± 0.02 ^b^	16.24 ± 0.12 ^a^
Kolbach index (%)	54.23 ± 1.40	-
α-amino nitrogen (mg/100 g)	144.40 ± 2.79	-
Saccharification power (WK)	108.40 ± 2.35	-
Original extract (°P)	-	8.80 ± 0.14
Alcohol (%vol)	-	3.28 ± 0.21
Soluble solids (%)	-	1.60 ± 0.11
Total acidity (mL/100 mL)	-	1.97 ± 0.12
Reducing sugar (mg/mL)	-	45.27 ± 0.25
Fermentation degree (%)	-	76.94 ± 5.38
Total phenolics (mg GAE/L)	-	250.45 ± 6.69
Total flavonoids (mg RE/L)	-	97.91 ± 3.05
ABTS scavenging rate (%)	-	87.41 ± 1.13
DPPH scavenging rate (%)	-	92.68 ± 1.90

Data are expressed as mean ± SD (n = 3). Different superscript letters in the same row indicate significant differences between K3 and K4 (independent-samples *t*-test, *p* < 0.05). Parameters not determined for a given stage or measured only in finished beer (K4) are marked as “-” and are presented as descriptive statistics (mean ± SD) without significance letters.

**Table 3:** Aroma-active compounds (ROAV ≥ 1) identified across different processing stages of black highland barley beer.

No.	Compound	Odor Description	Odor Threshold (µg/L)	ROAV (K1)	ROAV (K2)	ROAV (K3)	ROAV (K4)
1	Furfural	bread, almond	2.0–713	0.02	0.68	1.50	2.05
2	isoamyl alcohol	sweet, malty,	1.69–1750	0.47	0.70	0.14	71.59
3	2,3-Butanediol	fruity, onion	4.5	0.09	3.07	0.50	0.30
4	1-Hexanol	green grass, plastic	2.4–16,000	3.83	1.06	0.21	0.25
5	Nonanal	aldehyde, citrus, orange peel	1	0.22	2.08	0.24	0.01
6	Benzeneacetaldehyde	floral, honey	1	0.10	7.40	1.18	13.67
7	Acetic acid, 2-phenylethyl ester	rose, honey	108	0.00	0.00	0.00	1.67
8	Vanillin	vanilla, caramel, sweet	0.0002–92.9	100.00	100.00	100.00	37.80
9	Hexadecanoic acid, ethyl ester	Wax	2	0.17	0.26	0.02	5.08
10	Acetic acid, butyl ester	sweet, banana	0.13–368,000	3.99	14.58	0.52	0.36
11	Acetophenone	sweet, almond, pungent, oranges, river water	0.24–590	0.58	0.52	0.33	7.70
12	2-Undecanone	orange, fresh, green	0.0044	6.46	15.56	4.05	65.37
13	Limonene	lemon, plastic, citrus, rubber, terpene	1.8–310	2.32	3.27	0.00	0.03
14	2,3,5-Trimethylpyrazine	roasted nuts, cocoa, peanuts	0.023	2.93	2.04	45.84	22.20

Note: ROAVs were calculated using vanillin as the reference standard (ROAV = 100). Compounds with ROAV ≥ 1 are defined as aroma-active. Data are expressed as mean values of three biological replicates. Odor thresholds were retrieved from FlavorDB (https://cosylab.iiitd.edu.in/flavordb/, accessed on 20 July 2026).

**Table 4:** Flavoromics detection instruments.

Name	Brand	Model
Gas chromatography	Agilent (Santa Clara, CA, USA)	8890A
Mass spectrometer	LECO (St. Joseph, MI, USA)	Pegasus BT 4D
Ultra-High Performance Liquid Chromatography (UHPLC)	ExionLC AD	Sciex (Framingham, MA, USA)
High-Sensitivity Mass Spectrometer (HS-MS)	QTrap 6500+	Sciex
Centrifuge	Heraeus Fresco17	Thermo (Waltham, MA, USA)
Water Purification System	Clear D24 UV	Merck Millipore (Darmstadt, Germany)
Chromatographic Column	ACQUITY UPLC HSS T3 1.8 μm 2.1 × 100 mm	Waters (Milford, MA, USA)
DVB/CAR/PDMS (50/30 μm, 1 cm)	Supelco (Bellefonte, PA, USA)	57329-U

## Data Availability

The original contributions presented in the study are included in the article, further inquiries can be directed to the corresponding author.

## Supplementary Materials

The following supporting information can be downloaded at https://www.mdpi.com/article/10.3390/molecules31173062/s1: Table S1: ROAV Values of 77 Volatile Compounds Identified across K1–K4; Figure S1: Displays representative total ion chromatograms (TICs) from GC×GC-TOF MS analysis, where (A) presents the one-dimensional separation profile (1D-TIC) and (B) shows the orthogonal two-dimensional separation (2D-TIC). The x-axis denotes primary retention time (s), while the y-axis represents secondary retention time (s). The color gradient reflects signal intensity of detected ions, with red hues indicating higher response intensities and blue indicating lower intensities; Figure S2: Extracted Ion Chromatogram of QC Samples; Figure S3: Accumulation of representative phenolic and flavonoid compounds across the four processing stages of black highland barley beer (K1, raw grain; K2, germinated malt; K3, roasted malt; K4, finished beer).
