# Supplementary material for: Integrative Analysis of Widely Targeted Metabolomics and Flavoromics Reveals Dynamic Changes in Characteristic Metabolites at Different Processing Stages of Black Highland Barley Beer
Source: Molecules. 2026 Aug 31;31(17):3062. doi: 10.3390/molecules31173062 (PMC13567510; doi:10.3390/molecules31173062)
Supplement: Supplementary file 1 [file molecules-31-03062-s001.zip › Supplementary FigureS1-2.pdf]

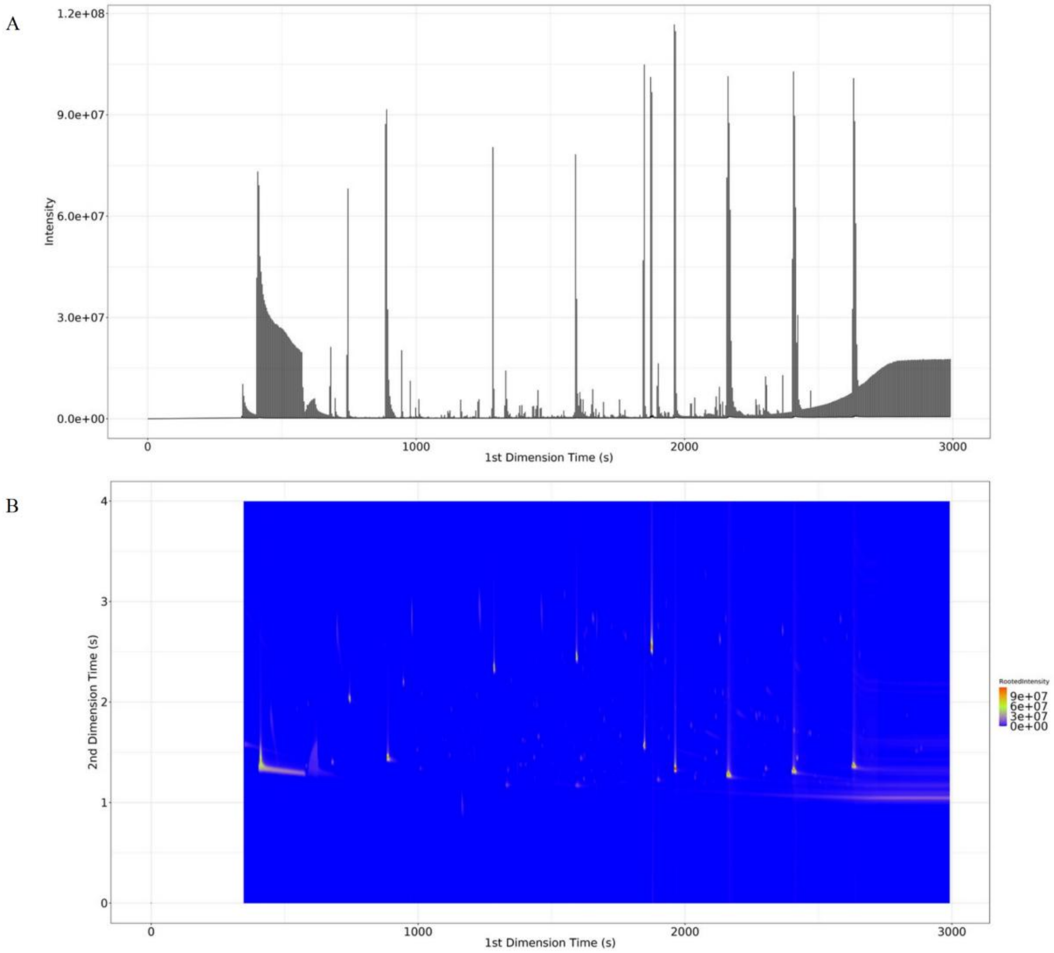

Fig. S1 Displays representative total ion chromatograms (TICs) from GC×GC-TOF MS analysis, where

(A) presents the one-dimensional separation profile (1D-TIC) and (B) shows the orthogonal two-dimensional separation (2D-TIC). The x-axis denotes primary retention time (s), while the y-axis represents secondary retention time (s). The color gradient reflects signal intensity of detected ions, with red hues indicating higher response intensities and blue indicating lower intensities.

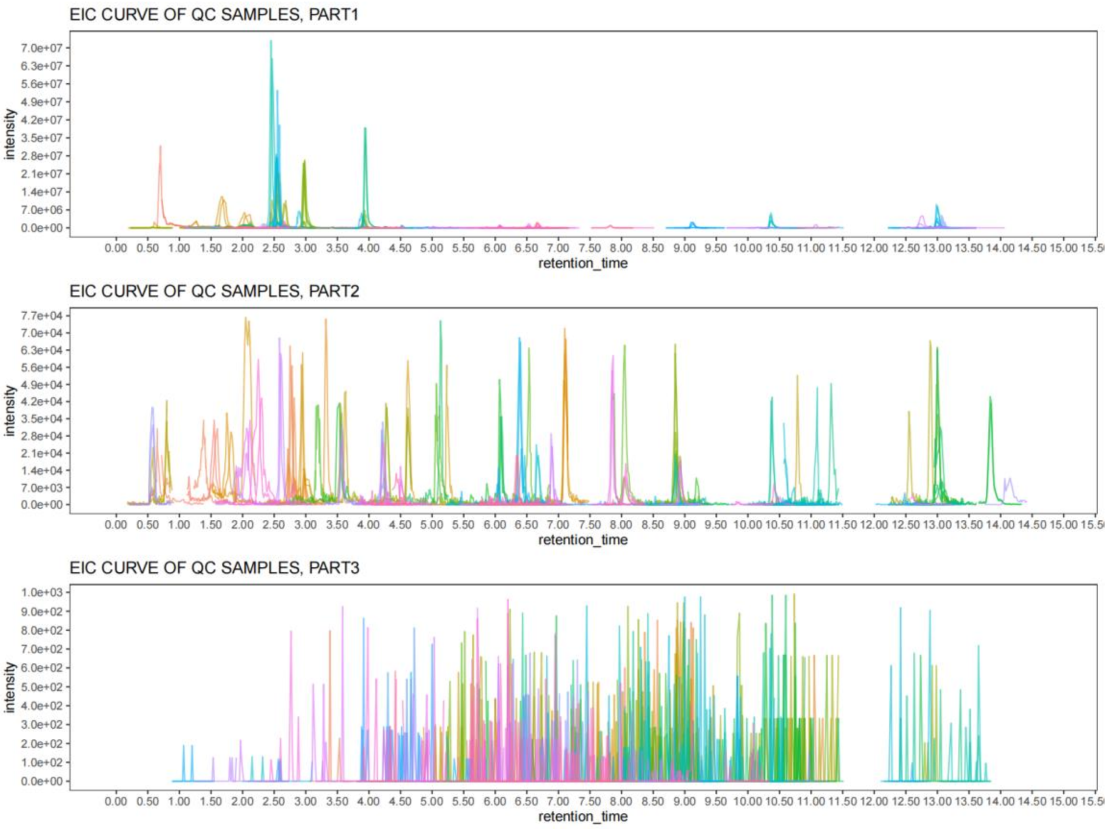

Fig. S2 Extracted Ion Chromatogram of QC Samples
