# Supplementary material for: Integrative Analysis of Widely Targeted Metabolomics and Flavoromics Reveals Dynamic Changes in Characteristic Metabolites at Different Processing Stages of Black Highland Barley Beer
Source: Molecules. 2026 Aug 31;31(17):3062. doi: 10.3390/molecules31173062 (PMC13567510; doi:10.3390/molecules31173062)
Supplement: Supplementary file 1 [file molecules-31-03062-s001.zip › Supplementary FigureS3.pdf]

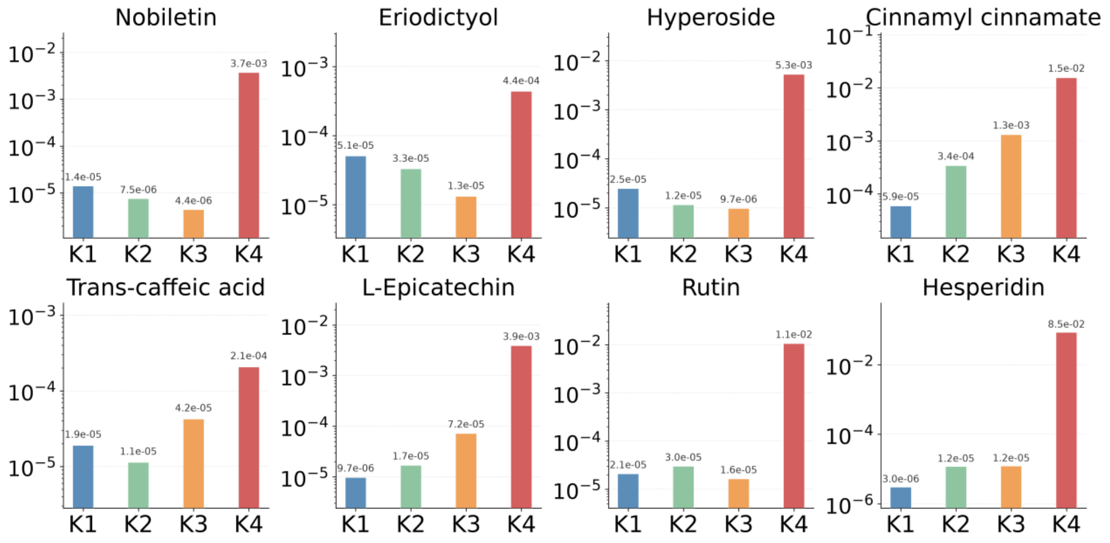

Fig. S3 Accumulation of representative phenolic and flavonoid compounds across the four processing stages of black highland barley beer (K1, raw grain; K2, germinated malt; K3, roasted malt; K4, finished beer).
